# Mechanisms for the formation and maintenance of microhabitat self-organization in the root system of orchid

**DOI:** 10.3389/fgene.2026.1809943

**Published:** 2026-09-29

**Authors:** Junyang Song, Xinying Hao, Jianfeng Chen, Shankang Wang

**Affiliations:** 1 College of Landscape Architecture and Art, Northwest A&F University, Yangling, China; 2 Shaanxi Shiquan Orchid Technology Co., Ltd, Shiquan, China

**Keywords:** orchid, rhizosphere soil, root, root microhabitat, self-organization, symbiotic microorganisms

## Abstract

Orchid species, globally valued for their ornamental qualities, serve as a model system for studying plant-microbe symbiosis. Most orchid species establish characteristic mycorrhizal associations, with their root microhabitats playing pivotal roles in host growth, development, and ecological adaptation through dynamic soil–plant–microbe interactions. Despite their ecological importance, orchid species remain underrepresented in symbiosis research compared to other plant. This paper reviews recent research advances on the formation and maintenance mechanisms of root microhabitat self-organization in orchids (Orchidaceae). The review covers the techniques and methodologies for orchid root microhabitat research, the relationship between orchid roots and rhizosphere soil, the relationship between orchid endophytic and rhizosphere microorganisms and rhizosphere soil, the relationship between orchid roots and endophytic and rhizosphere microorganisms, the tripartite interaction among orchid roots, rhizosphere soil and endophytic/rhizosphere microorganisms, as well as the relationship between root exudates, microbial metabolites and soil biofilms. The mechanisms underlying orchid root-soil-microorganism interactions are discussed. On this basis, the concept and relevant theories of orchid root microhabitat self-organization are proposed, and the formation and maintenance mechanisms of this self-organization process are elaborated. Finally, the prospects for future research on orchid root microhabitat self-organization are presented. This paper aims to provide references for the research on plant root microhabitat self-organization, as well as the conservation and utilization of orchid resources.

## Graphical Abstract

**Figure:**
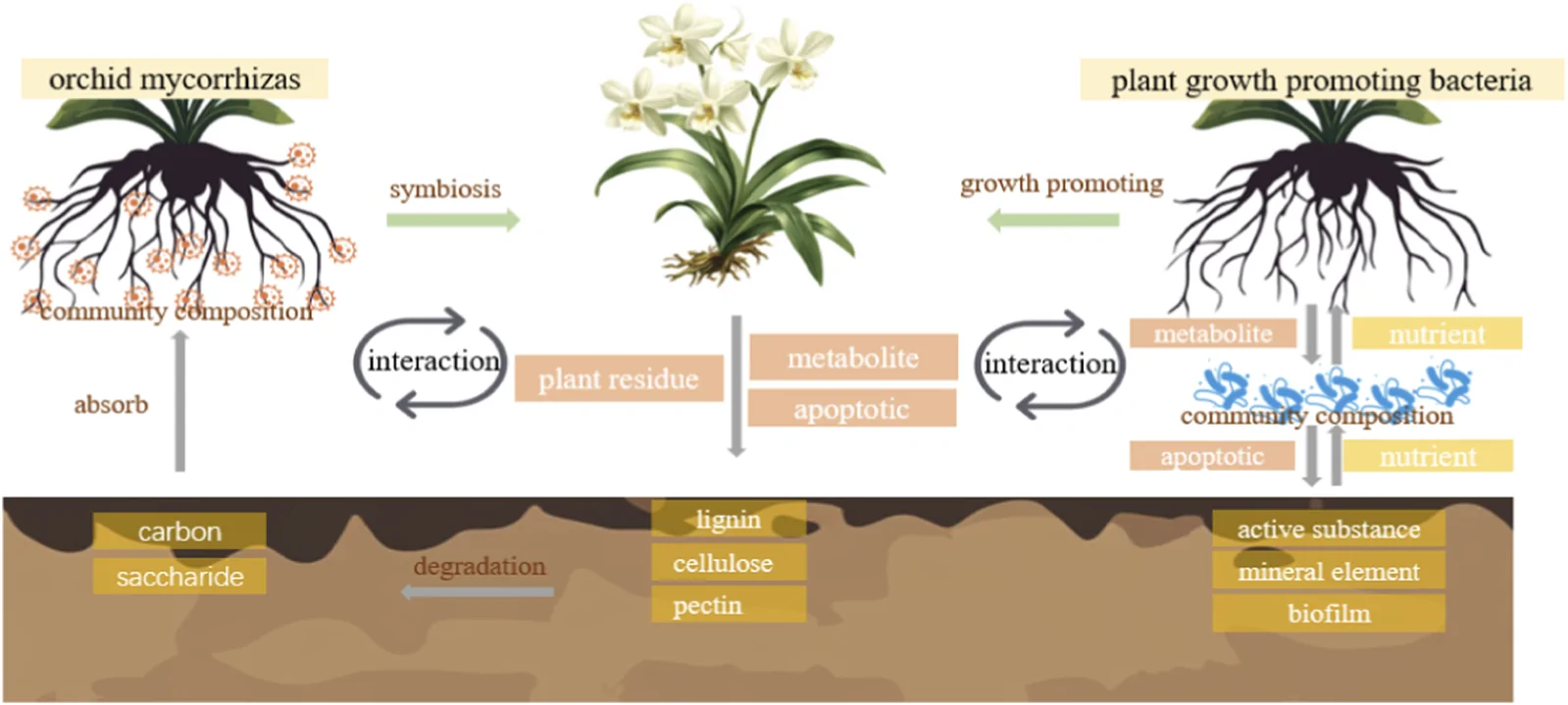

## Introduction

The Orchidaceae, a widely studied and taxonomically recognized family within the order Monocotyledons, exhibits remarkable biodiversity, encompassing thousands of described species distributed across diverse ecosystems worldwide. Demonstrating exceptional ecological plasticity, orchid species occupy a broad range of habitats (Wu et al., 2024), from tropical rainforests (Lan et al., 2025) to temperate zones (Gao et al., 2020) and xeric environments (Tao and Pei, 2024). However, anthropogenic habitat modification has precipitated significant declines in orchid diversity (Parra-Sanchez et al., 2024). The conversion of natural ecosystems has resulted in the fragmentation and outright loss of suitable orchid habitats, driving numerous species toward endangerment or extinction.

Orchid species exhibit remarkable reproductive diversity, employing both sexual and asexual propagation mechanisms (Huang et al., 2024). Sexual reproduction involves pollen dispersal and seed formation (Zhang M. et al., 2024), while asexual reproduction occurs through vegetative expansion, such as the proliferation of underground tubers or lateral branching. Notably, orchid seeds lack endosperm, rendering them highly dependent on external symbiotic interactions, particularly animal-mediated pollination and mycorrhizal associations—for successful germination and early development. Recent advances in high-throughput sequencing technologies have enabled the generation of comprehensive genomic, transcriptomic, and epigenomic datasets, providing unprecedented insights into orchid biology. These emerging molecular and multi-omics approaches (e.g., genomics, proteomics, metabolomics) are revolutionizing orchid breeding programs by accelerating trait discovery and genetic improvement (Li et al., 2021).

Root-associated microorganisms are integral to nutrient cycling and plant growth, forming dynamic microhabitats through complex tripartite interactions among host plants, microbial communities, and environmental factors. In orchid species, the rhizosphere microbiome comprises a highly specialized consortium of fungi, bacteria, actinomycetes, nematodes, and other soil-dwelling organisms, along with their surrounding edaphic ecological matrix. This microbiome exhibits remarkable complexity and host specificity, playing a critical role in orchid development, nutrient acquisition, and biotic interactions. Fungal symbionts—particularly mycorrhizal partners, are essential for seed germination and seedling establishment in orchid species, due to their obligate dependence on mycoheterotrophy during early life stages. Concurrently, bacterial and actinomycete communities contribute to nitrogen fixation, phosphate solubilization, and pathogen suppression, thereby enhancing host fitness. The obligate symbiosis between orchids and mycorrhizal fungi serves as the core survival foundation throughout their entire life cycle. Endosperm-free orchid seeds completely rely on obligate mycorrhizal fungi such as *Tulasnella* and *Sebacina* to supply carbon sources and mineral nutrients for protocorm establishment. After entering the autotrophic stage, mycorrhizas still continuously regulate the nutrient absorption efficiency of roots. Other associated microorganisms, including rhizosphere plant growth-promoting bacteria and endophytic actinomycetes, can enhance orchid stress resistance by secreting signaling molecules and assist in activating insoluble nutrients in the rhizosphere. These two types of symbionts synergistically participate in the self-organization construction of orchid rhizosphere microhabitats, and jointly support the complete nutrient acquisition process of orchids from seed germination to mature plant development. The intricate interplay between these microbial networks and orchid roots underscores their co-evolutionary adaptations, which are central to ecological resilience and niche specialization.

Orchids exhibit selectivity in microbial recruitment. Orchidaceae is a highly diverse plant family, and the potential host specificity of orchids drives different orchid taxa to recruit distinct microbial communities. This specific microbial recruitment trait of orchids leads to the formation of unique root rhizosphere microhabitats for different orchid species, and each microhabitat subsequently develops its own characteristic self-organization features. This is also the underlying mechanism that accounts for the remarkably diverse morphological phenotypes and species diversity of Orchidaceae in nature.

The symbiotic formation of mycorrhizae represents one of the most evolutionarily significant fungal adaptations to terrestrial ecosystems (Strullu-Derrien et al., 2018). In a novel microcosm experiment using field soil, Zhang C. et al. (2024) demonstrated that arbuscular mycorrhizal fungi (AMF) function synergistically with root systems to significantly enhance plant growth, nitrogen uptake, and mycorrhizal colonization rates. Their research further reveals the pivotal role of AMF in plant carbon allocation and soil organic matter (SOM) formation. AMF can directionally regulate the carbon allocation pattern of orchid hosts via hyphal networks, efficiently transport photosynthetic carbon to the rhizosphere to drive symbiotic interactions, and directly promote the stable formation of soil organic matter (SOM) through hyphal residues and metabolites. In nutrient-deficient habitats, this process continuously supplies limiting nutrients such as nitrogen and phosphorus for orchids, and substantially enhances the fitness and survivability of Orchidaceae in oligotrophic environments. -These findings hold profound implications for climate change mitigation and sustainable land management (Kakouridis et al., 2024). While root-associated microorganisms are fundamentally important for plant growth and health, current research has predominantly focused on agricultural and cash crops, leaving significant gaps in our understanding of other plant systems. Recent studies have shown that root exudates play a key role in shaping the rhizosphere of microbial communities. Flavonoids and strigolactones in orchid root exudates can directionally recruit obligate symbionts such as *Tulasnella* and *Sebacina*, while phenolic acids and specific terpenoid volatiles precisely repel pathogenic fungi and non-symbiotic saprophytic microbes. These sequentially secreted signaling compounds synergistically construct a root filtering network, which serves as the core chemical foundation driving the formation of ordered self-organized structures in the orchid rhizosphere microhabitat. Alahmad et al. (2024) found that root exudates of plant significantly influence the composition of root-associated microbiota and correlate with soil aggregation capacity, highlighting the complex interplay between plant secretions and microbial dynamics. Complementing this, Thoenen et al. (2023) showed that plant root bacteria exhibit specific tolerance to benzoxazinoids (BXs), suggesting that bacterial resistance to plant-derived anti-microbial compounds contributes to host-specific microbiome assembly. These findings collectively advance our understanding of plant–microbe–soil interactions in rhizosphere ecosystems.

Fungal communities, including saprophytic fungi and rhizosphere fungi, constitute vital components of orchid root microbiomes. These fungi form mutualistic relationships with orchid species, significantly enhancing host growth and nutrient acquisition (Cao, 2023). Concurrently, bacterial and actinomycete populations contribute critically to soil nutrient cycling and biocontrol processes. Mycorrhizal symbiosis—a widespread ecological phenomenon—represents a specialized mutualism between soil-inhabiting fungi and vascular plant root systems (Wang et al., 2021). Alibrandi et al. (2020) employed 16S rRNA macrobarcoding to analyze endophytic bacterial communities in three Mediterranean orchid species, identifying 1,930 operational taxonomic units (OTUs). Dominated by Proteobacteria and Actinobacteria, these communities exhibited consistent distribution patterns across root organs, suggesting that plant tissue type—rather than host species—primarily governs bacterial assembly. These findings indicate that orchid roots selectively recruit and maintain specialized bacterial consortia that contribute functionally to host physiology. Research on temperate terrestrial orchid species reveals that orchid mycorrhizal fungi (OMF) and other root-associated beneficial fungi are predominantly from the phyla Ascomycota and Basidiomycota, with distinct community compositions observed between native and plantation forests (Herrera et al., 2022). In a study of *Calanthe sieboldii*, Huang et al. (2022) demonstrated through diversity and eco-functional analyses that fungal communities exhibit spatial and temporal heterogeneity across root microhabitats. The endophytic fungal community proximal to orchid roots remained relatively stable yet showed seasonal dynamism, whereas distal soil fungi displayed greater variability. Notably, dominant endophytic fungi exhibited higher compositional stability than soil fungi, underscoring their critical role in orchid growth and development.

Orchid root microhabitats exhibit significant variation across environmental conditions, influenced by factors such as soil type, moisture availability, and light exposure. Through interactions with root-associated microorganisms, orchid species actively modulate soil microbial communities to enhance environmental adaptability. Collectively, orchid root systems constitute a sophisticated microecosystem that plays a critical role in supporting host plant growth, ecological adaptation, and broader ecosystem functions, including the maintenance of soil health and overall ecosystem stability.

Soil microbial biofilms refer to structured microbial aggregates that form on the surfaces of soil particles, comprising bacteria, fungi, actinomycetes, and other microorganisms. These communities produce extracellular polymeric substances (EPS) and biological adhesives that facilitate their attachment to soil particles, ultimately developing into a viscous thin-film structure. Research conducted by the State Key Laboratory of Agricultural Microbiology at Huazhong Agricultural University revealed that, while biofilm-associated and free-living soil communities share similar microbial compositions, biofilms exhibit significantly greater biodiversity and community evenness (Wu et al., 2019). Microorganisms within these biofilms engage in complex interaction networks with plant root systems, participating in key physiological processes such as nutrient acquisition (e.g., phosphate solubilization, nitrogen fixation), growth and development regulation (via phytohormone modulation), and enhanced stress tolerance (through induced systemic resistance and antioxidant production).

Soil biofilms exhibit dual ecological functions. On the one hand, they provide microenvironmental protection for microbial communities. On the other hand, they mediate the adhesion between microbial communities and soil particles. These structured assemblages enhance microbial metabolic activity, facilitate nutrient cycling, and contribute to soil aggregate stabilization, thereby promoting soil ecosystem resilience. Notably, biofilms in paddy fields function as biogeochemical reactors, converting atmospheric CO_2_ to CH_4_ and influencing greenhouse gas emissions (Wang et al., 2022). Furthermore, recent evidence identifies soil biofilms as hotspots for the horizontal transfer of antibiotic resistance genes (ARGs), underscoring their underappreciated role in ARG dissemination within soil ecosystems (Wu et al., 2022). These findings significantly advance our understanding of microbial functional dynamics in soil environments.

Orchid root-associated biofilms represent specialized microhabitats where Ascomycota and Basidiomycota fungi dominate, forming mycorrhizal structures essential for nutrient acquisition—particularly nitrogen and phosphorus—in nutrient-poor soils. These biofilms exhibit host-specific recruitment patterns, suggesting co-evolutionary relationships. However, the relative contributions of bacterial and fungal consortia to biofilm functionality remain poorly quantified, highlighting the need for metabolomics approaches to elucidate these complex interactions.

## Research techniques and methodologies for orchid root rhizosphere microhabitats

### Methods for the study of root microbial diversity

Analyzing soil microbial diversity in root microhabitats offers valuable insights into plant-soil-microbe interactions and the functional roles of rhizosphere microorganisms. Current approaches for studying soil microbial diversity primarily include biochemical methods (e.g., culture-dependent identification and substrate utilization assays) and molecular techniques (e.g., nucleic acid hybridization, soil metagenomics, and DNA polymorphism analysis) (Ma, 2019) (Table 1). These methodologies have been widely applied in orchid root microbiome research. Traditional culture-based methods allow for the direct isolation of microbial strains and the characterization of their physiological and ecological properties under controlled conditions. However, these approaches are limited by the inability to culture many soil microorganisms under artificial conditions. In contrast, molecular techniques such as 16S rRNA gene sequencing and metagenomics bypass the need for cultivation by extracting and sequencing DNA directly from environmental samples, enabling comprehensive profiling of microbial communities. While molecular methods effectively reveal microbial diversity, they lack functional characterization capabilities and may be influenced by PCR primer biases and sequencing errors. Culture-based methods remain superior for studying microbial physiology (Shi et al., 2023), whereas molecular approaches excel in analyzing community structure (Yang et al., 2024). Therefore, an integrated strategy that combines both culture-dependent and molecular techniques is recommended for a comprehensive investigation of orchid root-associated microorganisms, providing both functional and phylogenetic insights.

**TABLE 1:** Common approaches for microbial diversity analysis.

Subject/Study system	Method	Key findings	Reference
*Endophytic bacteria in Mediterranean orchid roots*	16S	Characterized endophytic bacterial community diversity and composition in orchid roots	Alibrandi et al., 2020
*Mycorrhizal fungi associated with Orchidaceae roots*	ITS	Identified 40 putative orchid mycorrhizal fungal taxa from root samples	Jacquemyn et al., 2020
*Rhizosphere microbial community and DOM composition*	HRM	Revealed diversity of DOM molecular composition and microbial community structure in rhizosphere soil	Lv et al., 2024

16S, 16S rRNA, gene metabarcoding; ITS, internal transcribed spacer sequencing; HRM, high-resolution mass spectrometry coupled with high-throughput sequencing.

### Methods of soil biological characteristics

Assessment of soil biological properties in rhizosphere studies typically involves evaluating microbial diversity and functional traits to elucidate the ecological roles of root microhabitats in soil ecosystems. This approach provides critical insights into plant-soil interactions and ecosystem stability. Modern investigations often integrate molecular techniques (e.g., high-throughput sequencing) with biochemical analyses to examine rhizosphere microbial enzyme activities, metabolic pathways, and byproducts. These combined methodologies enhance our understanding of microbial contributions to nutrient cycling, organic matter decomposition, and plant pathogen suppression in soil-based systems (Table 2). To elucidate root-microbe interactions and microbial community dynamics, researchers typically examine rhizosphere microbial communities and their functional traits by exploring the interplay between root exudates and microbial metabolites. Such assessments provide a scientific basis for understanding how rhizosphere microhabitats influence soil ecosystem functioning and stability.

**TABLE 2:** Common approaches for soil biological properties.

Subject/Study system	Method	Key findings	Reference
*Soil enzyme activities under microplastic exposure*	SPA	Demonstrated that microplastics significantly stimulated urease and phosphatase activities and altered soil bacterial community composition	Lian et al., 2024
*Microbial metabolite dynamics and soil chemistry*	RMD	Elucidated composition and spatiotemporal dynamics of microbial metabolites at fine-scale soil chemistry resolution	Wiesenbauer et al., 2024
*Root exudate metabolic profiles and soil microbial communities*	CHR	Characterized effects of root exudates on soil and synthetic microbial community composition and function	Basak et al., 2023

SPA, spectrophotometric analysis; RMD, reverse microdialysis combined with stable isotope tracing; CHR, chromatographic-based metabolic profiling.

### Methods of root microhabitat physiological ecology

Plants regulate microbial community structure and function through root exudates, including mucilage and volatile organic compounds. In return, soil microorganisms support plant growth by providing nutrients and bioactive compounds via organic matter decomposition and nitrogen fixation. root system architecture (such as length, diameter, surface area, and spatial distribution) serves as a key indicator of plant-microbe interactions. A large number of studies collectively investigate root growth, rhizosphere enzyme activities, genetic regulation of root development, and plant responses to environmental stress using various physiological and ecological approaches (Table 3). Plants enhance stress resilience through coordinated modulation of root architecture, exudation patterns, and metabolic activity. Overall, plant physiological ecology fundamentally reflects root-environment interactions and plays a critical role in rhizosphere studies. Investigating root system architecture, functional traits, and stress response mechanisms provides key insights into plant-soil interactions and helps elucidate the ecological adaptation strategies that govern belowground plant systems.

**TABLE 3:** Common approaches for root physiological ecology.

Subject/Study system	Method	Key findings	Reference
*Root architectural responses toheterogeneous soil structure*	IMA	Quantified root growth and architectural adaptation patterns under altered soil physical structure conditions	Giuliani et al., 2024
*Spatiotemporal enzyme activity and exudation patterns in rhizosphere*	ZYM	Mapped spatial glucose release patterns and β-glucosidase/leucine aminopeptidase activity dynamics in the rhizosphere	Kaloterakis et al., 2024
*Molecular regulation of root growth and defense signaling*	MOL	Identified PSKR1-mediated regulatory pathway controlling root growth, photosynthesis, and salicylic acid defense responses	Song et al., 2023
*Rhizosphere microbiome and root exudates under salt stress*	IMA	Characterized variation in rhizosphere/endophytic bacterial communities and root exudate profiles across salt tolerance gradients	Cao et al., 2024

IMA, image-based root phenotyping and architecture analysis; ZYM, zymography for spatial enzyme activity imaging; MOL, molecular and transcriptional analysis.

### Review of research methods in root microhabitat

Since orchid roots are located underground, the research on orchid root rhizosphere microhabitats is constrained by numerous technical and methodological limitations. The existing techniques for orchid root rhizosphere research have multiple inherent defects. First, the spatiotemporal resolution of *in-situ* observation techniques is severely insufficient. Traditional rhizosphere researches rely on destructive rhizobox sampling, subsequent isolation and culture, or universal amplicon sequencing. These methods cannot capture the millimeter-scale or even micron-scale dynamics of the microhabitat without disturbing the *in-situ* symbiotic state of orchid roots and mycorrhizas, completely losing the spatial heterogeneity information of rhizosphere biofilms and hyphal networks, and failing to accurately verify the spatially ordered structure of orchid rhizosphere self-organization. Second, the capacity for coupled analysis of abiotic-biotic interactions is absent. Most existing techniques can only separately measure rhizosphere physicochemical factors, microbial communities or root physiological indicators, and cannot synchronously track the dynamic cascading relationships among root exudates, microbial necromass and microbial community metabolic activity, making it difficult to directly confirm the core scientific hypothesis that “root vital activities are the core driving factor of self-organization”. Third, deviations exist in the functional interpretation of orchid-specific symbiosis. Universal metagenomic techniques show extremely low functional annotation coverage for orchid-specific low-abundance symbiotic fungi (*Tulasnella*, *Sebacina*), and a large number of unique metabolic pathways of orchid mycorrhizas cannot be accurately identified, resulting in the failure to distinguish general rhizosphere processes from the orchid-specific self-organization driving processes. Last but not least, the *in vitro* experimental systems are severely disconnected from the original habitat. Traditional *in vitro* culture systems for mycorrhizal symbiosis completely ignore the *in-situ* dynamic conditions such as rhizosphere soil biofilms and natural alternating drying-wetting cycles, and the obtained interaction rules cannot restore the self-organization evolution process in real orchid root rhizosphere microhabitats.

With the deepening of relevant researches and the advancement of science and technology, existing techniques and methodologies have been greatly improved and upgraded, and a series of brand-new techniques and methods have also emerged.

For the *in-situ* rhizosphere visualization observation technology, it adopts the latest non-invasive planar optode coupled with micro-scale fluorescence imaging technology. Without any disturbance to the microhabitat, this technology can perform real-time dynamic monitoring on the micron-scale spatial distribution of oxygen, pH and nutrient concentrations in the orchid rhizosphere, while tracking the colonization dynamics of labeled symbiotic fungal hyphae in the biofilm on the root surface, so as to precisely capture the formation process of the ordered self-organized structure.

For the coupled *in-situ* metabolomics-single-cell technology, it leverages spatial metabolomics to directly locate the distribution of root exudates and microbial metabolites in different rhizosphere micro-regions. Combined with single-cell sorting technology, it can directly target and isolate target symbiotic fungal cells, resolve their metabolic activity at the single-cell level, and accurately establish the direct coupling relationship of “root vital activity - metabolite distribution - microbial community function”.

For the orchid-specific functional gene capture technology, it designs targeted capture probes based on the published database of orchid mycorrhizal fungi-specific functional genes, to specifically enrich the functional sequences of *Tulasnella* and *Sebacina* from the complex rhizosphere metagenome. This technology can drastically improve the annotation accuracy of orchid-specific symbiotic metabolic pathways and solve the annotation deviation problem of universal omics techniques.

For the microfluidic rhizosphere chip simulation system, it uses a customized orchid rhizosphere microfluidic chip to precisely simulate the *in-situ* dynamics of nutrient fluctuation and drying-wetting alternation. Meanwhile, it enables real-time observation on the complete self-organization process of orchid seed-symbiotic fungus interaction and biofilm formation at the micron scale, completely eliminating the defect that the *in vitro* culture system is severely disconnected from the original habitat.

### Self-organization of orchid root rhizosphere microhabitats

#### Relationship between roots and soil

The survival and reproduction of plants are fundamentally affected by their surrounding environments. Global vegetation can be roughly divided into two major categories. One is natural ecosystems with minimal human activities and anthropogenic interventions, which is the core research domain of ecology. The other is agroecosystems with intense artificial interventions, which falls into the research scope of agronomy. Ecological researches mainly explore the relationships between the distribution, survival and reproduction of plants in natural environments and corresponding environmental factors, while agronomic researches focus on investigating the growth and development of crops under artificial cultivation conditions. Most studies on plant-environment interactions prioritize the status of above-ground plant parts, such as the growth vigor and biomass of wild plants, as well as the yield and quality of crops. The research contents include the role of plants in ecosystem functions and community succession (Winberg et al., 2024; Xie et al., 2024; Garcia-Figuera et al., 2024), the adaptive responses of plants to extreme climates (Wang Y. et al., 2024; Yuan et al., 2024), and the regulatory mechanisms of plant photosynthesis, light signal transduction and target trait genes in crops (Liu M. et al., 2023; Rahman et al., 2024).

Restricted by conventional research habits and technical equipment, human beings have extremely limited research and exploration on the below-ground parts of plants. The status and processes occurring in plant below-ground parts still remain an unknown territory. The main component of plant below-ground parts is the root system. The performance of plant above-ground parts largely depends on the plant root system: the above-ground part is only the external manifestation of the vital activities of plant roots, while the root system is the most critical and essential organ of plants (Song, 2017). As the main organ for plants to acquire resources and interact with the environment, the root system functions as a central regulatory hub. Roots absorb inorganic nutrients (e.g., nitrogen, phosphorus, potassium) and organic compounds (e.g., carbohydrates, lipids, phytohormones) required for growth and development (Masuda et al., 2024), and coordinate the substance synthesis, energy allocation and signal transduction within plants.

Apart from its role in nutrient acquisition, the root system can significantly affect the physical, chemical and biological properties of the surrounding soil (Table 4). The growth and development of roots will alter soil structure, porosity, stability and hydrological regimes. Root exudates released by roots can change soil pH value and EC value, and shape the composition and function of microbial communities.

**TABLE 4:** The role of the plant root system on the soil.

Root trait	Soil property	Relationships	Literature
Root density	Saturated hydraulic conductivity (Ks)	Nonlinear (hump-shaped)	Ma et al., 2024
Root density	Saturated water content (SW)	Nonlinear (hump-shaped)	Ma et al., 2024
Root surface area	Soil separability	Negative	Goet et al., 2024
Root-activated C	Natural soil N	Positive	Song et al., 2024
Root N content	Soil P supply	Subtle	Guilbeault-Mayers and Laliberté, 2024
Root area	Alkaline cations	Positive	Staszel-Szlachta et al., 2024
Root length	Soil enzyme activity	Positive	Staszel-Szlachta et al., 2024
Root apoplast	Soil fauna abundance	Subtle	Zhou et al., 2023
Root apoplast	soil porosity	Positive	Uteau et al., 2022
Root exudates	Soil structure	Positive	Baumert et al., 2021
Root exudates	Persistent soil carbon (PSC)	Positive	Gregory, 2022

Relationship between microorganisms and soil.

These multifunctional roles establish roots as key mediators in plant-soil feedback systems. Most plants are soil-dependent, with their root systems intricately interacting with the soil environment. Brunello et al. (2024) demonstrated that *Phalaenopsis* roots exhibit photosynthetic activity, producing oxygen under light exposure to alleviate hypoxia in root tissues. Orchid roots also display specialized adaptations that support survival under specific soil conditions, where favorable edaphic factors are critical to their growth. Plant physiological processes are tightly coupled with root function, which is, in turn, governed by both direct and indirect interactions with the soil environment (Zou et al., 2024). Collectively, root-soil microenvironments play pivotal roles in modulating the environment and sustaining plant vitality.

#### Relationship between microorganisms and soil

Soil ecosystems host a wide array of living organisms, including plant roots, soil fauna and microorganisms. Among these, soil microorganisms represent a critical biological resource, playing indispensable roles in soil formation and ecosystem functioning. Despite being largely invisible, their vast species and genetic diversity has become a central focus in biodiversity research (Ma et al., 2023). Advances in metagenomics have revolutionized our understanding of soil microbial communities, providing powerful tools to decode their complexity (Tyson et al., 2004). Soil microbial communities fundamentally affect the physicochemical properties of soil through multiple pathways (Table 5). These effects include mutualistic interactions characterized by dynamic feedback mechanisms, and soil microbe-driven decomposition and transformation of organic matter, which further facilitate the release of nutrients and regulate the cycles of carbon, nitrogen and phosphorus within the soil ecosystem. Moreover, they shape the community structure, diversity and functional activity of microorganisms via ecological functions including competition, symbiosis and synergy.

**TABLE 5:** The relationship between soil properties and microorganisms.

Microbial group	Microorganisms	Soil property	Relationships	Literature
Bacteria	*Pseudomonas aeruginosa*	Al toxicity	Negative	Castrillo et al., 2017
	*Rhodococcus erythropolis*	Al toxicity	Negative	Castrillo et al., 2017
	*Bacillus subtilis*	Soil acidity	Positive	Rudrappa et al., 2008
	*Nocardia*	SOC conversion	Positive	Zhang D. et al., 2024
	*Pseudomonas aeruginosa*	SOC conversion	Positive	Zhang D. et al., 2024
	*Pseudomonas aeruginosa*	Urease & dehydrogenase activities	Positive	Yu et al., 2024
	*Bacillus subtilis*	Urease & dehydrogenase activities	Positive	Yu et al., 2024
Fungi	Proteobacteria	Metabolic versatility	Positive	Yang et al., 2023
	Acidobacteria	Metabolic versatility	Negative	Yang et al., 2023
	Pontospora	Metabolic versatility	Negative	Yang et al., 2023
	Blastomonas	Metabolic versatility	Negative	Yang et al., 2023
	Chlorobacteria	N_2_O emission	Negative	Xu et al., 2024
	*Actinomyces*	N_2_O emission	Negative	Xu et al., 2024
	Ascomycetes	Metabolic versatility	Negative	Yang et al., 2023
	Basidiomycota	Metabolic versatility	Unrelated	Yang et al., 2023
	Actinobacteria	N_2_O emission	Negative	Xu et al., 2024

A global study across 20 distinct soil ecosystems found that microbial biodiversity and ecological networks are sustained through positive inter-domain associations (e.g., among bacteria, protists, fungi, and invertebrates), with tripartite symbiotic relationships particularly common. These findings underscore the role of cooperative interactions in maintaining ecosystem functioning (Liu et al., 2024). Targeted soil microbiome engineering has emerged as a promising strategy for addressing soil degradation and environmental challenges by harnessing microbe-mediated modifications of soil properties (Philippot et al., 2023).

Microbial metabolic secretions act as key mediators of species interactions in soil ecosystems. Recent metabolic modeling studies have shown that cost-free metabolites can modify the soil environment and trigger interspecific relationships, while also quantifying the relative contributions of environmental factors to these processes (Pacheco et al., 2019). Metabolic exchange is a fundamental feature of microbial communities, with cross-feeding interactions being especially prevalent. These specialized, often synergistic metabolic relationships underpin global biogeochemical cycles, particularly those involved in organic matter decomposition. Although obligate metabolic interactions frequently exhibit synergy (Kost et al., 2023), their detection remains methodologically challenging due to the limitations of laboratory systems in replicating the complexity of natural environments.

Soil microorganisms also play a pivotal role in carbon sequestration and global carbon cycling (Chen et al., 2024). Researchers at the U.S. Department of Energy’s Berkeley Lab recently developed a novel genomics-informed model that enhances understanding of microbial-mediated carbon sequestration (Marschmann et al., 2024). In a complementary effort, Xu et al. (2023) examined soils from 180 sites across China using metagenomic sequencing, high-throughput qPCR, and culture-based approaches (Dai et al., 2023). Their results revealed that trace elements and macronutrients (C, N, P) interact in a cogwheel-like mechanism to shape microbial community structure and function. Notably, Wang X. et al. (2024) demonstrated that microbial metabolic limitations governed by C: N: P stoichiometry promote the accumulation of soil organic carbon (SOC) in agricultural systems, offering new insights into carbon storage strategies. Recent studies have further elucidated that microorganisms exhibit a dual regulatory mechanism in soil organic carbon dynamics, which balances the carbon allocation between assimilation for growth and carbon loss and consumption via respiration (Tian et al., 2024). However, the relationship between microbial carbon use efficiency and SOC persistence remains complex and insufficiently explored, marking a critical knowledge gap in terrestrial carbon cycling research.

Soil biofilms, which are composed of bacteria, fungi and protozoa, form microbial aggregates on the soil surface or within the soil matrix. These biofilms secrete adhesive extracellular polymeric substances, stabilize soil structure and regulate key biogeochemical processes. A growing body of evidence has demonstrated that soil biofilms support diverse microbial communities and enhance their metabolic activities (Wu et al., 2019). Microbial residues significantly affect soil ecosystem functions and biodiversity. Research from China Agricultural University shows that fungal residues can enhance soil aggregation and organic matter stability, both of which are key factors determining the quality of agricultural soils (Liu L. et al., 2023). Networks formed by fungi also play a pivotal role in regulating the assembly of root-associated microbial communities, and driving the spatial heterogeneity and resource use efficiency in agroecosystems (Zhou et al., 2020).

While microbiome research has made notable progress in agricultural settings, studies in native soil ecosystems remain comparatively scarce (Li et al., 2023), highlighting an important gap in our understanding of soil microbial ecology across diverse land-use types.

#### Relationship between microorganisms and roots

Nature’s remarkable complexity and interdependence are profoundly expressed below ground. Plant roots and their associated microorganisms form intricate symbiotic relationships that go beyond mere coexistence, encompassing sophisticated nutrient cycling and biochemical signaling (Ivanov and Harrison, 2024). The specific plant signaling molecules therein play a pivotal role in mediating the microbial recruitment and community assembly in the orchid rhizosphere. These interactions are fundamental to the survival and ecological success of both partners.

In root–microbe–soil ecosystems, roots act as dominant drivers of these relationships by actively selecting and shaping surrounding microbial communities. This root-mediated assembly process preferentially recruits microorganisms that confer adaptive advantages (Zhang Y. H. et al., 2024). The structure and activity of microbial communities significantly influence the physicochemical properties of soil. Latest advances in mycorrhizal research indicate that mycorrhizal hyphal networks connect plant roots, regulate nutrient fluxes, and mediate interplant interactions. These are all key processes that shape the ecology of plant communities (Liu et al., 2024). The root crown plays a pivotal role in rhizosphere microbial aggregation (Rüger et al., 2023), with conserved colonization patterns observed across plant species (Vannier et al., 2023). However, the molecular mechanisms underlying microbial symbiont activation and host colonization remain poorly understood. Technological breakthroughs have begun to unravel these mechanisms. Yu et al. (2023b) identified key regulators of mycorrhizal symbiosis, and Bell et al. (2024) mapped a Y1H-based regulatory network linking transcription factors such as PHRs to symbiosis-related genes through P1BS elements.

Plant metabolites are crucial mediators of plant–microbe interactions, affecting development, stress responses, and overall plant health. Recent studies have shown that root exudates can reshape rhizosphere bacterial communities and enhance phosphorus uptake (Wang et al., 2023). Groundbreaking research has also revealed that arbuscular mycorrhizal fungi may use a parasitic strategy to redirect host carbon allocation toward colonized roots (Bell et al., 2024), suggesting a potentially widespread symbiotic mechanism. Additionally, microbial-derived volatile organic compounds (VOCs) in the rhizosphere significantly influence plant biomass and agronomic traits (Ma et al., 2023). root-derived methyl jasmonate (MeJA), for example, functions as a bioactive VOC that induces rapid changes in both the rhizosphere microbiome and plant epidermis (Kulkarni et al., 2023).

Current researches mainly focus on how symbiotic microorganisms improve nutrient acquisition of food crops and cash crops. However, compared with wild plants, cultivated plants usually exhibit lower nutrient use efficiency, which is caused by long-term artificial selection of cultivated plants in eutrophic environments (Oldroyd and Leyser, 2020). These findings highlight the potential of applying the symbiosis principle between wild plants and microorganisms to improve crop nutrient use efficiency, so as to reduce the reliance on synthetic and organic fertilizers.

Among microorganisms, fungi are the most widely studied and prevalent symbiotic partners of plant roots. In a comprehensive review, Bonfante and colleagues (Genre et al., 2020) explored the evolutionary origins of mycorrhizal fungi, their host range, and their potential biotechnological applications, emphasizing both conserved and lineage-specific aspects of mycorrhizal symbiosis. Studying root–fungal associations poses technical challenges due to the subterranean nature of root systems (Song, 2017). To address this, researchers at Cambridge developed an innovative *in vivo* visualization system using betalain pigments, enabling real-time, non-invasive monitoring of arbuscular mycorrhizal colonization (Timoneda et al., 2021). While traditional staining techniques allow symbiotic visualization, manual detection of root complexity remains subjective and labor-intensive. To overcome this, Evangelisti et al. (2021) developed an automated computer vision system based on convolutional neural networks to quantify AM fungal colonization and intraradical mycelial structures.

Research on orchid root–microbe interactions remain limited (Lv et al., 2023; Xu et al., 2023) (Table 6). However, orchid species are known to form mycorrhizal associations with fungi from families such as Ceratobasidiaceae and Tulasnellaceae, which enhance host nutrition by facilitating the transfer of phosphorus, nitrogen, and micronutrients. In addition, rhizosphere microorganisms contribute to orchid growth indirectly by decomposing organic matter in the soil, thereby improving nutrient availability.

**TABLE 6:** Orchid root systems and microorganisms.

Orchid species	Microorganism family	Microbial group	Functional role	Literature
*Epipactis palustris*	Serendipitaceae	Endophytic fungi	Nutrient delivery	Zahn et al., 2023
	Ceratobasidiaceae	Endophytic fungi	Nutrient delivery	Zahn et al., 2023
	Tulasnellaceae	Endophytic fungi	Nutrient delivery	Zahn et al., 2023
*Dactylorhiza fuchsii* var. *rhodochila*	Ceratobasidiaceae	Endophytic fungi	Carbon transfer	Read et al., 2024
*Dendrobium officinale*	Amphisphaeriaceae	Endophytic fungi	Growth promotion	Tao, 2024
	Nectriaceae	Endophytic fungi	Growth promotion	Tao, 2024
*Spiranthes sinensis*	Ceratobasidiaceae	Endophytic fungi	Fungible carbon supply	Yukari et al., 2014
*Epipactis atrorubens*	Ascomycota	Rhizosphere fungi	Nutrient source	Zahn et al., 2023
	Basidiomycota	Rhizosphere fungi	Nutrient source	Zahn et al., 2023

#### Root-soil-microorganism relationships

Plant root microhabitats host complex root–soil–microbe interactions that collectively maintain a dynamic, self-regulating equilibrium (Figure 1). roots and soil exhibit an interdependent relationship. While roots absorb essential nutrients for plant growth, they simultaneously alter the physical properties of soil (Siqueira et al., 2023) and shape microbial community composition (Yu et al., 2023a; Mueller et al., 2024). microorganisms play a pivotal role in this system by establishing symbiotic relationships with roots. Regulating plant responses to abiotic stresses and diseases via rhizosphere signal transduction has been a prominent research hotspot in recent years. Through rhizosphere signaling, they facilitate nutrient and phytohormone exchange, thereby enhancing plant growth and resistance to pathogens (Tong et al., 2024). Additionally, microbial-mediated decomposition of organic matter releases plant-available nutrients, improving soil fertility (Liu M. et al., 2023). Environmental factors such as soil pH, salinity, and moisture significantly regulate root–microbe interactions by influencing microbial growth and metabolic activity (Sokol et al., 2024; Lange et al., 2024).

**FIGURE 1:**
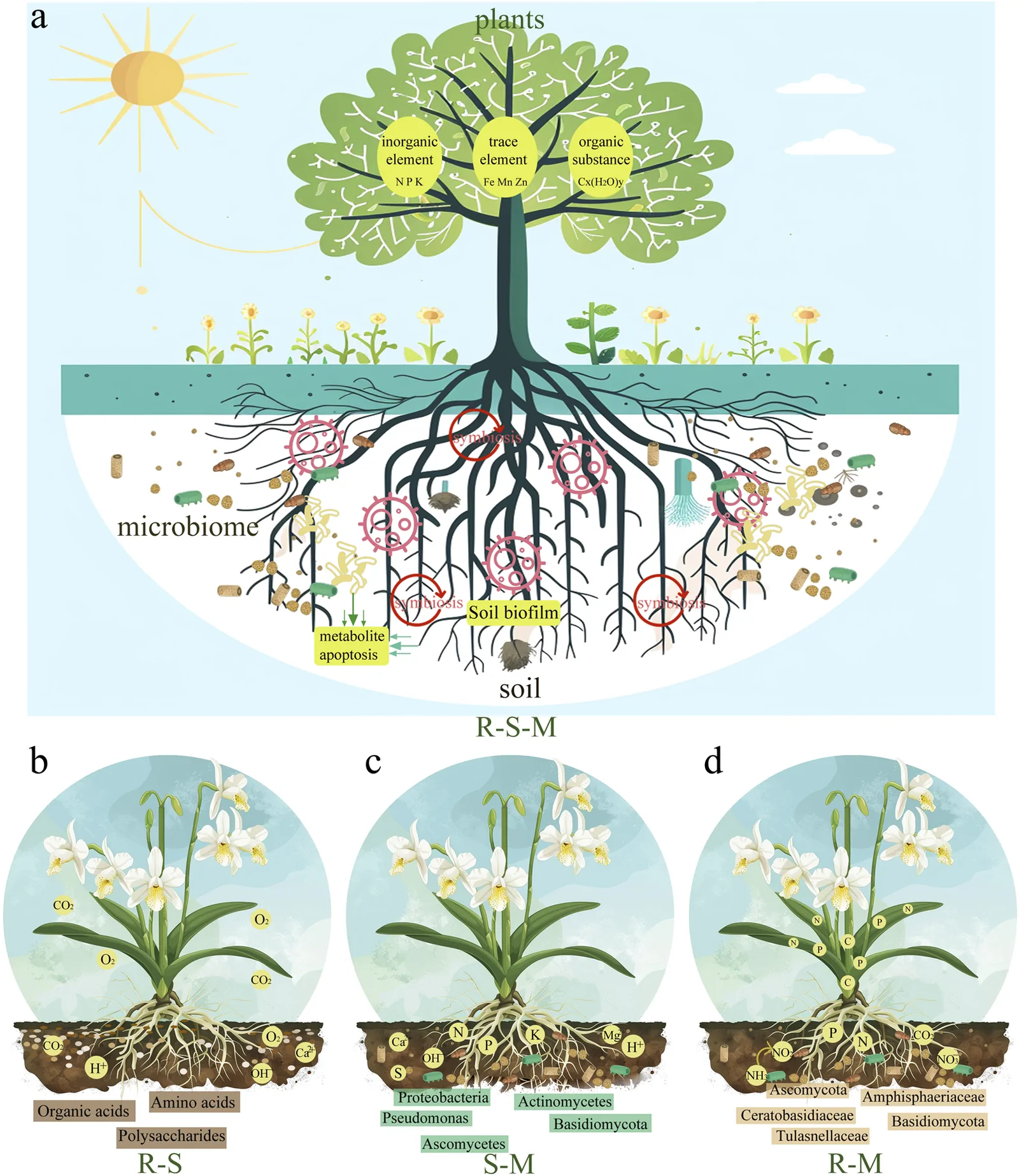
Root-soil-microbe relationships. **(a)** Root-soil-microorganism relationships; **(b)** The relationship between the root system and the soil; **(c)** The relationship between soil and microorganisms; **(d)** The relationship between root systems and microorganisms. R-root; S-soil; M-microorganisms.

The micro (local living growth ground) of orchid roots is characterized by a complex and diverse community of fungal and microbial associates that play crucial roles in orchid ecology and physiology. Zelmer et al. (1996) highlighted the association of fungi with terrestrial orchid mycorrhizas, seeds, and protocorms, emphasizing the importance of fungal partners during early developmental stages. Similarly, Cameron et al. (2006) provided evidence for mutualistic interactions in orchid mycorrhiza, demonstrating bidirectional carbon transfer and organic nitrogen acquisition, thereby underscoring the functional significance of these fungal associations within the orchid root micro (Cameron et al., 2006).

Yuan et al. (2009) further elaborated on the diversity and specificity of fungal communities associated with cherries, reporting the widespread presence of non-mycorrhizal endophytic fungi in both aboveground and belowground tissues of epiphytic orchid species. Their findings suggest tissue-specific assemblages and spatial heterogeneity in endophyte communities, indicating that the micro encompasses a complex network beyond traditional mycorrhizal fungi. Expanding on this, Huang et al. (2022) investigated the spatiotemporal dynamics of fungal taxa within the root micro of *Calanthe sieboldii*, offering insights into the functional roles and community structure of fungi across time and space—knowledge vital for understanding ecological stability and informing conservation strategies.

Research on orchid-associated bacteria has also contributed to our understanding of the microhabitat. For example, Tsavkelova et al. (2007) identified bacterial strains that produce growth-promoting substances such as indole-3-acetic acid, which can influence root development and seed germination. These microbial interactions further highlight the micro as a nexus of diverse microbial life that supports orchid growth and propagation.

Overall, the rhizosphere microbial environment is a dynamic and ecologically rich system. Existing studies have demonstrated that there exist close and complex interactions among the root system, soil and microorganisms within this environment (Figure 1). The orchid root micro is a dynamic and ecologically rich environment composed of mycorrhizal fungi, endophytes, and bacteria that collectively influence orchid development, health, and ecological interactions. The reviewed studies underscore the importance of microbial diversity, tissue specificity, and temporal dynamics in shaping the micro of orchid roots, offering a foundation for future ecological and conservation research. The three components form a tightly coupled interaction network that critically regulates plant growth and development, ecological adaptation mechanisms and soil ecosystem multifunctionality. Plant roots mediate bidirectional matter and energy exchange with the rhizosphere through chemical exudation (e.g., organic acids, amino acids, polysaccharides) and morphological plasticity (root architecture modulation). The rhizosphere soil serves as the primary interface for plant-microbe interactions. Key soil components including organic matter, nutrient elements, microbial communities, and bioactive compounds. They play critical roles in regulating root growth and microbial metabolic activities. Endophytic microorganisms in root including bacteria, fungi, and actinomycetes, establish symbiotic relationships with plant roots. These microorganisms participate in critical soil processes such as nutrient cycling, biological nitrogen fixation, organic matter decomposition, and plant pathogen suppression. Additionally, they modulate soil bioactive compounds, regulate enzymatic activities, and enhance soil aggregate formation, thereby influencing the physical, chemical, and biological properties of the rhizosphere soil.

#### Self-organization in the rhizosphere microhabitat of orchids

Self-organization theory is an interdisciplinary theory that studies complex systems spontaneously evolving from disorder to order through internal mechanisms without specific external instructions, with its core being revealing the law of systems forming ordered structures through self-driving. If a system acquires spatial, temporal or functional structures without specific external intervention, this process belongs to a self-organization process.

The orchid root rhizosphere microhabitat consists of the orchid root system, the microorganisms residing inside the root tissues and in the soil surrounding the roots, as well as the rhizosphere soil around the root system. The orchid root rhizosphere microhabitat is a self-organizing system. In this microhabitat, the components with life characteristics are the root system and microorganisms. The vital activities of the root system and microorganisms are the power source for the existence and maintenance of this microhabitat, among which the vital activities of the root system act as the main power source.

According to the above-mentioned self-organization theory, first of all, a system that generates self-organization must be an open system. The orchid root rhizosphere microhabitat is an open system. Existing studies have proved that there are exchanges of matter, energy and information in the microhabitat of orchid root-microorganism-soil. Secondly, the system must be far from the equilibrium state when developing from disorder to order. Orchids continuously input carbon sources and energy into the microhabitat by secreting rhizodeposits such as organic acids and sugars through the root system, accompanied by the dynamic inflow and outflow of water and mineral elements, so that the system can never reach the thermodynamic equilibrium state. The continuous metabolic activities of orchid mycorrhizal fungi, which decompose organic matter and synthesize phytohormones, constantly break the local nutrient steady state, keeping the nutrient concentration and microbial community abundance in the rhizosphere always with significant spatial heterogeneity, far away from the uniform equilibrium state. The dynamic changes of cultivation environment such as alternating dry and wet conditions and temperature fluctuations continuously impact the original steady state of the rhizosphere, preventing the system from falling into an inactive equilibrium dead state, and providing a necessary prerequisite for the formation of self-organized ordered structures. Finally, there are nonlinear interactions among the subsystems within the system. Existing research results show that there are nonlinear interactions between orchid roots and symbiotic fungi, nonlinear synergy among orchid rhizosphere microbial communities, and nonlinear feedback in the coupling of orchid root-soil-microorganism.

As a rigorously defined ecological term, the microhabitat, characterized by dynamic ontogeny and deterministic community assembly traits, has been extensively validated across a large body of empirical studies. Current research efforts predominantly focus on discrete biological self-organization phenomena within microhabitat systems, while the construct of “microhabitat self-organization” has not yet been formalized as a standalone, widely recognized proprietary academic concept in the field. The above review summarizes the research findings on the microhabitat of orchid roots in recent years. It elaborates the relationship between orchid roots and rhizosphere soil, the relationship between endophytic and rhizosphere microorganisms of orchids and rhizosphere soil, the relationship between endophytic and rhizosphere microorganisms of orchids and orchid roots, as well as the tripartite interaction relationship among orchid roots, rhizosphere soil and microorganisms. On this basis, this dissertation proposes the concept of self-organization of orchid microhabitat.

Self-organization of orchid root microhabitat refers to an ecological evolution process that occurs in the rhizosphere micro-domain dominated by orchid roots. Driven by the vital activities of roots and microorganisms, it relies on the targeted regulation of root exudates, synergistic interactions of rhizosphere microbial communities, and dynamic adaptation of micro-environmental physicochemical factors. Without external artificial intervention, this process spontaneously forms a specialized microhabitat system with ordered structure and functional homeostasis. During this process, orchid roots recruit functional microbial groups by secreting specific metabolites. Mycorrhizal fungi and beneficial rhizosphere microorganisms expand nutrient acquisition niches through hyphal networks, gradually constructing a micro-ecosystem with self-regulation, efficient nutrient transformation, and active stress resistance. This ultimately achieves synergistic adaptation among orchid roots, microorganisms and rhizosphere soil, representing a core ecological strategy evolved by orchids over long-term evolution to maintain nutrient acquisition and habitat stability.

### Perspectives on root microhabitat research in orchidaceae

#### Root system characteristics of orchidaceae plants and the microbial characteristics within and in the rhizosphere

Most terrestrial plants form symbiotic relationships with soil microorganisms, and the orchidaceae represent a classic model for studying fungal symbiosis (Wang et al., 2021). Our long-term ecological studies of wild *Cymbidium* populations in China’s Qinling Mountains have revealed distinct spatial patterning in their aboveground distributions. This observation raises fundamental questions about their belowground microbial ecology. Do orchid root systems exhibit similar spatial organization in their associations with microbials? What factors govern the composition and diversity of endophytic versus rhizospheric microbial communities? Through what mechanisms do orchid roots interact with their associated microbiota? And how have these root–microbe systems coevolved over time? Addressing these questions requires a comprehensive characterization of orchid root system architecture, alongside detailed taxonomic and functional profiling of root-associated microorganisms. Equally important is elucidating the dynamic interactions between orchid species and their microbial partners to better understand the ecological and evolutionary underpinnings of these complex symbiosis.

Orchidaceae encompass a remarkably high diversity of species. Terrestrial orchids and epiphytic orchids exhibit drastically different growth habits, which generate substantial divergence in their selective pressure on microorganisms, and further lead to distinct differences in the formation and maintenance mechanisms of their microhabitat self-organization. The theory of orchid root rhizosphere microhabitat self-organization can drive orchids to directionally recruit specific symbiotic microorganisms, precisely shape their exclusive rhizosphere niche, and substantially enhance the ecological resilience of the rhizosphere micro-system. For instance, in oligotrophic rocky habitats, this self-organization mechanism can rapidly reconstruct the mycorrhizal symbiosis network, and support *Paphiopedilum* to stably complete seed germination and population persistence in nutrient-deficient cliff habitats.

#### The spatiotemporal characteristics of root secretions and microbial metabolites in the root microhabitat of orchidaceae plants

The orchid root rhizosphere microhabitat is composed of three components: the orchid root system, the microorganisms residing inside root tissues and in the surrounding soil, as well as the rhizosphere soil around the root system. The tangible solid entities in this system are roots, microorganisms and soil particles, while the remaining components are various organic or inorganic chemical substances in liquid, gaseous or ionic states. Within this system, the components with life characteristics and vital activities are the root system and microorganisms. Root exudates are the primary products of root vital activities, and microbial metabolites are the main products of microbial vital activities.

The orchid root rhizosphere microhabitat represents a dynamic interface, where living entities (roots and microorganisms) and non-living components (soil particles) interact via various chemical substances, jointly modulating plant growth and development. This complex system has emerged as a central focus in contemporary agricultural and ecological research. Conventional studies generally prioritize the physicochemical properties of soil, such as texture, moisture, temperature, nutrient availability, gas composition and pH value (Sheikh et al., 2024; Ziskin et al., 2024; Zhou et al., 2024). However, we propose that root exudates and microbial metabolites should be recognized as the most critical substances in the orchid root rhizosphere microenvironment. This is because these substances are the products of vital activities (metabolism) of the living entities (roots and microorganisms) within this microhabitat, and act as the pivotal mediators for plant-microbe-soil interactions. Investigations on the spatiotemporal characteristics of orchid root exudates and microbial metabolites should be established as the core priority of orchid root rhizosphere microhabitat research. The research on orchid root exudates and microbial metabolites should focus on the relationships between key vital activities of roots, including generation, development and apoptosis, and these substances. We hypothesize that the dominant factor governing the self-organization of orchid root rhizosphere microhabitat is likely the vital activities of the root system, while the community assembly and metabolic activities of microorganisms serve the vital activities of roots. In this process, sophisticated material exchange, energy flow and information communication are embedded throughout the whole system.

#### Characteristics of soil biofilms in the rhizosphere of orchidaceae and their relationship with microbial apoptosis

While biofilm research has traditionally focused on medical and environmental engineering applications, investigations on soil biofilms remain relatively understudied (Hou et al., 2024). In the plant root rhizosphere microhabitat, biofilms primarily colonize the root surface and adjacent rhizosphere soil, serving as a critical hub for microbial ecological processes. Accumulating evidence demonstrates that the formation and functionality of biofilms are tightly linked to microbial programmed cell death. Notably, recent studies have revealed that microbial necromass plays a pivotal role in the structural assembly of the rhizosphere microenvironment and the functional persistence of these ecological niches.

The entire life cycle of orchids is inextricably dependent on microorganisms, especially at the initial stage of orchid life, i.e., seed germination. According to self-organization theory, evolutionary pressure is an essential prerequisite for a self-organizing system to develop from a disordered state to an ordered state. Orchid seeds lack endosperm, and their germination obligately relies on heterotrophic nutrition supplied by specific fungi. Soil biofilms act as an indispensable medium for microbial colonization on orchid seeds. Recent studies have revealed that the formation of soil biofilms is associated with the apoptosis of roots and microorganisms. These findings raise two fundamental research questions regarding the self-organization of orchid root rhizosphere microhabitats: Does microbial necromass participate in the formation of rhizosphere biofilms? What role does it play in regulating energy and material flows in the orchid rhizosphere microenvironment? These unresolved questions constitute one of the core research priorities for future investigations on the self-organization of orchid root rhizosphere microhabitats.

#### The uniqueness of orchid-microorganism interactions and the applicability of self-organization theory in orchid root rhizosphere microhabitats

The high dependency of orchids on mycorrhizal fungi and their unique specificity in fungal recruitment represent the core symbiotic characteristics that distinguish Orchidaceae from other plant taxa. Orchid seeds are completely devoid of endosperm nutrient reserves, and rely entirely on specific mycorrhizal fungi to supply carbon sources, mineral nutrients and growth regulators throughout the whole process from protocorm formation at the germination stage to subsequent seedling establishment, forming an obligate heterotrophic symbiosis. Even after entering the autotrophic growth stage, the vast majority of orchids still cannot maintain normal root physiological functions without their symbiotic fungal partners. This full-life-cycle obligate dependency is extremely rare among higher plants.

Orchid roots do not randomly recruit ubiquitous environmental microorganisms. Instead, they perform targeted recruitment of orchid-specific symbiotic fungal taxa dominated by *Tulasnella* and *Sebacina* via specific signaling molecules in root exudates, while actively repelling the colonization of non-functional miscellaneous microbes. This process ultimately forms a highly conserved endophytic community structure inside the roots. Such a precise host filtering mechanism is one of the core driving links for the formation of ordered self-organized structures in the orchid rhizosphere microhabitat.

Anthropogenically driven loss of habitat environmental heterogeneity directly disrupts the *in-situ* homeostasis of orchid rhizosphere biofilms, blocks the host filtering pathway by which orchid roots directionally recruit obligate symbiotic fungi via specific signaling molecules, disturbs the metabolic cascade coupling between abiotic and biotic rhizosphere components, and impedes the formation and functional maintenance of ordered self-organized structures in the rhizosphere microhabitat. The theory of orchid root rhizosphere microhabitat self-organization breaks through the traditional orchid conservation research paradigm that exclusively focuses on individual plants, and provides original theoretical support for the *in-situ* conservation of Orchidaceae, population reintroduction and reconstruction, and mycorrhizal symbiosis functional restoration from the novel dimension of rhizosphere system self-organization evolution.

The root rhizosphere microhabitat of Orchidaceae consists of three core components: the root system, soil medium and microbial community. Within this system, the living entities are roots and microorganisms, while the non-living substances are primarily produced and driven by the vital activities of roots and root-associated microorganisms. These non-living substances include root metabolites (especially root exudates), microbial metabolites, post-mortem residues of roots and microorganisms, various enzymes, natural soil mineral elements, and so on. These non-living substances are closely associated with the vital activities of the two living entities (roots and microorganisms). Complex dynamic relationships exist among them, accompanied by self-organization phenomena. The formation and maintenance of such self-organization revolve around the vital activities of orchid roots. Ultimately, the interaction between abiotic substances and the vital activities of organisms in the orchid rhizosphere microhabitat is the core scientific issue in this research field. The present thesis proposes the theory of root rhizosphere microhabitat self-organization for Orchidaceae, and whether this theory is applicable to other plant species remains to be further research.
